# F-Box Protein FBXO22 Mediates Polyubiquitination and Degradation of CD147 to Reverse Cisplatin Resistance of Tumor Cells

**DOI:** 10.3390/ijms18010212

**Published:** 2017-01-20

**Authors:** Bo Wu, Zhen-Yu Liu, Jian Cui, Xiang-Min Yang, Lin Jing, Yang Zhou, Zhi-Nan Chen, Jian-Li Jiang

**Affiliations:** National Translational Science Center for Molecular Medicine, Cell Engineering Research Center & Department of Cell Biology, State Key Laboratory of Cancer Biology, Fourth Military Medical University, 169 Changle West Road, Xi’an 710032, China; soldier2158wubo@163.com (B.W.); qqqzhenyu20052008@163.com (Z.-Y.L.); cuij93@163.com (J.C.); yxiangmind@163.com (X.-M.Y.); jinglin314@163.com (L.J.); 15209262969@163.com (Y.Z.)

**Keywords:** CD147, FBXO22, ubiquitination, tumor, chemo-resistance

## Abstract

Drug resistance remains a major clinical obstacle to successful treatment of cancer. As posttranslational modification is becoming widely recognized to affect the function of oncoproteins, targeting specific posttranslational protein modification provides an attractive strategy for anticancer drug development. CD147 is a transmembrane glycoprotein contributing to chemo-resistance of cancer cells in a variety of human malignancies. Ubiquitination is an important posttranslational modification mediating protein degradation. Degradation of oncoproteins, CD147 included, emerges as an attractive alternative for tumor inhibition. However, the ubiquitination of CD147 remains elusive. Here in this study, we found that deletion of the CD147 intracellular domain (CD147-ICD) prolonged the half-life of CD147 in HEK293T cells, and we identified that CD147-ICD interacts with FBXO22 using mass spectrometry and Western blot. Then, we demonstrated that FBXO22 mediates the polyubiquitination and degradation of CD147 by recognizing CD147-ICD. While knocking down of FBXO22 prolonged the half-life of CD147 in HEK293T cells, we found that FBXO22 regulates CD147 protein turnover in SMMC-7721, Huh-7 and A549 cells. Moreover, we found that the low level of FBXO22 contributes to the accumulation of CD147 and thereafter the cisplatin resistance of A549/DDP cells. To conclude, our study demonstrated that FBXO22 mediated the polyubiquitination and degradation of CD147 by interacting with CD147-ICD, and CD147 polyubiquitination by FBXO22 reversed cisplatin resistance of tumor cells.

## 1. Introduction

Chemotherapy presently is the core of anticancer treatment, and the development of targeted cancer therapies has advanced the treatment of various cancers with improved antitumor efficacy and specificity. Nonetheless, drug resistance remains a major clinical obstacle to successful treatment of resistant tumors [1]. The drug resistance is proposed to be facilitated by a small number of pre-existing cancer cells that are intrinsically resistant or poised to adapt to drug treatment quickly and also responsible for tumor initiation, progression, relapse and metastasis, resulting in tumor recurrence [2,3].

CD147 (EMMPRIN, basigin or HAb 18G) is a ubiquitously expressed glycoprotein that is commonly over-expressed in many tumors and plays critical roles in tumor progression [4,5]. In particular, CD147 has been strongly implicated in the chemo-resistance of cancer cells [6,7]. Protein modification often controls many important aspects of protein activity, localization, and stability. Numerous posttranslational protein modifications including glycosylation, ubiquitination, and even phosphorylation are reported to regulate the expression and function of CD147 [5,8,9]. Our previous study demonstrated that *N*-glycosylation of CD147 was remarkable in the inducing of matrix metalloproteinase activity. CD147 could also be internalized through an ARF6 (ADP-ribosylation factor 6) -related clathrin-independent endocytosis (CIE) pathway, and recycle back to the cell membrane with the mediation of hook1, Rab22a and microtubules [10,11,12]. The endocytosed cargoes can also be trafficked to the multivesicular bodies for lysosomal degradation [13]. Actually, a balance between ubiquitination and deubiquitination exists in the cell that determines the fate of internalized proteins: recycling to the membrane versus trafficking to the lysosome [14]. The plasma membrane levels of channels and receptors are often rapidly regulated by alterations of their trafficking itinerary [14]. Ubiquitination of CD147 seems to play a critical role in determining the cell membrane expression level and thereafter the function of CD147. For instance, TRAF6 regulates melanoma invasion and metastasis through ubiquitination of CD147 [9]. However, the mechanism and significance of CD147 ubiquitination remains elusive.

FBXO22 is a novel characterized F-box protein mediating the degradation of KDM4A (Lysine (K)-specific demethylase 4A), KLF4 (Kruppel-like factor 4) and methylated p53 [15,16,17]. Classically, F-box protein together with Skp1 and Cullin 1 constitute Skp-Cullin-F box ubiquitin E3 ligase (SCFs), which function in phosphorylation-dependent ubiquitination. However, the biological function of FBXO22 remains largely unknown. In this study, we first demonstrated that FBXO22 polyubiquitinated the internalized CD147 leading to its degradation. Moreover, we found that the low level of FBXO22 contributes to the accumulation of CD147 and thereafter the cisplatin resistance of A549/DDP cells.

## 2. Results

### 2.1. Ectopic CD147 Avoids Degradation after Intracellular Domain Deletion

**Figure 1:**
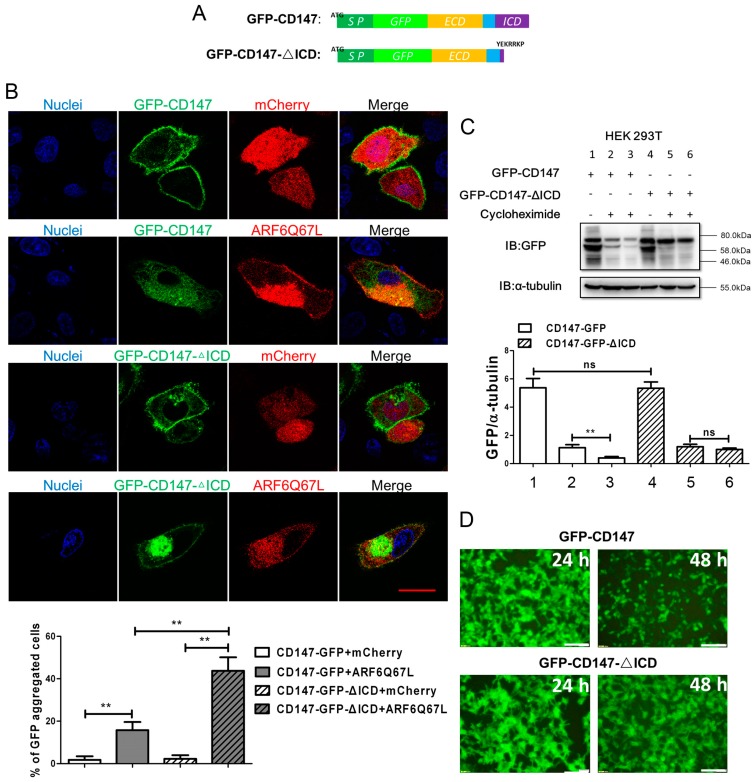
Deletion of intracellular domain enhances the stability of ectopic CD147. (**A**) Schematic representation of different constructs of CD147 inserted with GFP (structures showed in the upper panel, SP: signal peptides, green; ECD: extracellular domain, yellow; transmembrane domain, blue; ICD: intracellular domain); (**B**) representative distribution of exogenous GFP-CD147 or GFP-CD147-ΔICD co-transfected with mCherry/ARFQ67L-mCherry in SMMC-7721 cells. Growing SMMC-7721 cells were transfected with indicated plasmids for 36 h. Exogenous GFP-CD147 or GFP-CD147-ΔICD was expressed on the membrane but accumulated in the cytoplasm when ARFQ67L was co-transfected. DAPI (4′,6-diamidino-2-phenylindole) staining was used to visualize nuclei. The percentage of GFP aggregated cells was determined by scoring >100 cells. The data are the mean of the “% of GFP aggregated cells” from three independent experiments ± SD (Scale bar = 20 μm) (**C**) constructs of CD147 were first transfected into HEK293T cells separately. After transfection for 24 h, cycloheximide (final concentration: 100 μg/mL) was added into indicated samples. Cells in lanes 1, 2, 4 and 5 were collected 24 h later. Cells in lanes 3 and 6 were cultured for another 24 h. Finally, all of the samples were lysed with RIPA (Radio-Immunoprecipitation Assay) lysis buffer and analyzed by Western blot. Bottom: Western blot scanning densitometry for three independent experiments. Blots were probed for α-tubulin to ensure equal protein loading. ns = none significance; ** *p* < 0.01; (**D**) representative images showing expression level of transfected exogenous GFP-CD147 or GFP-CD147-ΔICD after cycloheximide treatment for indicated time. (Scale bar = 500 μm).

Typically, CD147 is distributed on the cell membrane, and membrane CD147 could be internalized through an ARF6-related CIE pathway [12]. Exposed to cytoplasm, the intracellular domain of CD147 (CD147-ICD) is indispensable for CD147 recycling back to the cell membrane. The sequential endocytosis and recycle of CD147 is proposed to be a tightly regulated progress involving ARF6 (ADP-ribosylation factor 6), hook1, Rab22a and microtubules [10,18]. Transfection of ARF6Q67L-mCherry, a constitutively activated form of ARF6, induced the formation of special structures termed vacuolar membranes. Membrane proteins internalized through ARF6-related CIE pathway accumulated in this special structure. As shown in Figure 1A, we first fused the full length CD147 with GFP to create GFP-CD147, and then the coding sequence of CD147-ICD was deleted to create GFP-CD147-ΔICD. Both products of GFP-CD147 and GFP-CD147-ΔICD were localized to the membrane when co-transfected with mCherry, but accumulated in cytoplasm when co-transfected with ARF6Q67L-mCherry (Figure 1B). Considering normal ARF6 exists in the transfected cells at a basal level, the recycling of GFP-CD147 was not fully disrupted. However, deletion of CD147-ICD in GFP-CD147 further enhances GFP-CD147-ΔICD accumulation in the cytoplasm, implying the critical role of CD147-ICD for CD147 recycling. Cargoes internalized into cytoplasm generally avoid degradation by vesicle recycling, which is an important process is for cells’ efficient operation [19]. Therefore, without intracellular domain, GFP-CD147-ΔICD was thought to be more readily degraded. To our surprise, GFP-CD147-ΔICD was in fact more stable than GFP-CD147, or the half-life of GFP-CD147-ΔICD was longer than GFP-CD147 in HEK293T cells (Figure 1C,D). Actually, there exists a balance between ubiquitination and deubiquitination dominating the destiny of internalized proteins [14]. Here, the unstable state of GFP-CD147 might result from its ubiquitination. To conclude, CD147-ICD is independent for CD147 internalization, but may mediate its degradation by ubiquitination.

### 2.2. CD147-ICD Interacts with FBXO22

**Figure 2:**
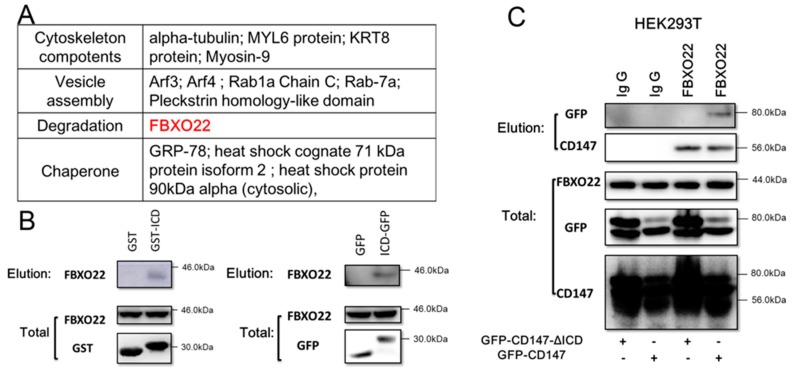
CD147-ICD interacts with FBXO22. (**A**) Mass spectrometry analyses showed potential proteins interacting with CD147-ICD, and FBXO22 (red) is a potential molecular medicating the degradation of CD147; (**B**) Western blot confirmed CD147-ICD interacts with FBXO22 (left). After eGFP-C2-ICD (ICD-GFP) was overexpressed in SMMC-7721 cells for 36 h, cells were lysed and collected onto a coupling resin pre-bound with GFP-antibody. Eluted samples were then applied to Western blot (right); (**C**) after GFP-CD147-ΔICD or GFP-CD147 was overexpressed in HEK293T cells for 36 h, cells were lysed and collected onto a coupling resin pre-bound with Ig G or FBXO22 antibody as indicated. Eluted samples were then applied to Western blot to detect GFP or CD147.

The ubiquitination of CD147 has been studied previously [9,20] but remains obscure. Moreover, ubiquitination is a complex process consisting of three enzyme groups: E1, E2 and E3. This cascade regulates the addition of ubiquitin moieties to specific proteins within a cell, leading to protein degradation. As CD147-ICD was hypothesized to mediate CD147 degradation by ubiquitination, we then performed IP (Immunoprecipitation)-mass spectrometry with GST-CD147-ICD (prokaryotic expression protein) to further identify the degradation process of CD147. As shown in Figure 2A, FBXO22 was identified. The interaction between GST-CD147-ICD and FBXO22 was further confirmed by Western blot (Figure 2B, left). Moreover, the interaction between CD147-ICD and FBXO22 was confirmed in eukaryotic protein system with ICD-GFP (Figure 2B, right). The interaction of FBXO22 with CD147 via CD147-ICD was further confirmed in Figure 2C.

### 2.3. FBXO22 Mediates Poly-Ubiquitination and Degradation of CD147

**Figure 3:**
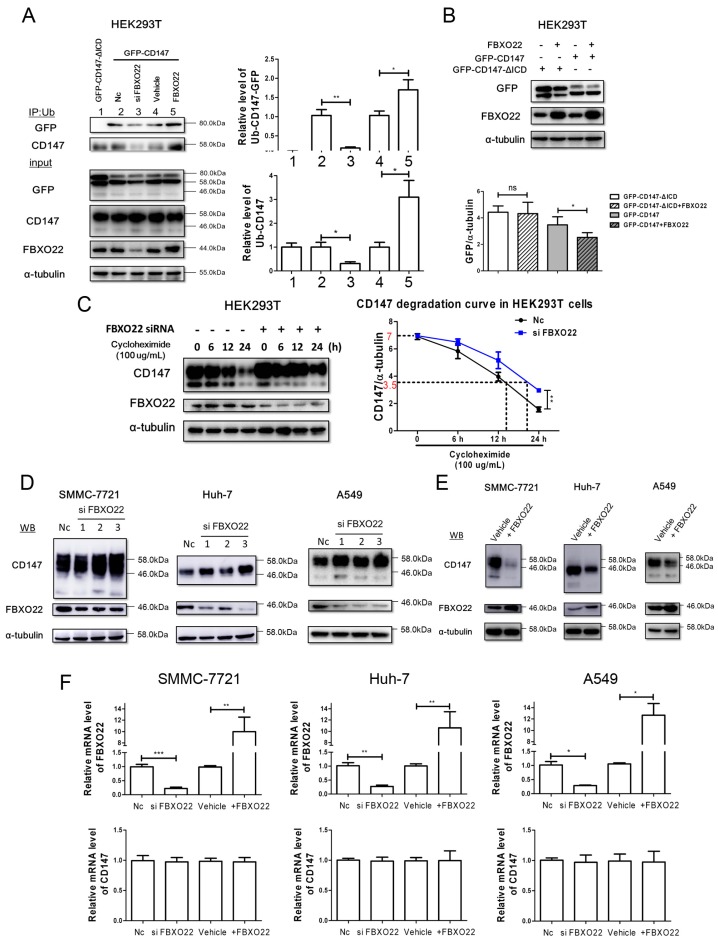
FBXO22 mediates poly-ubiquitination and degradation of CD147. (**A**) FBXO22 mediated the polyubiquitination of GFP-CD147. Vectors expressing the indicated proteins were transiently transfected into HEK293T cells. At 40 h after transfection, cell lysis was immunoprecipitated with anti-Ub antibody. Immune complexes were blotted with the indicated antibodies. Western blot scanning densitometry for three independent experiments. * *p* < 0.05, ** *p* < 0.01; (**B**) ectopic expression of FBXO22 destabilizes GFP-CD147, but not GFP-CD147-ΔICD. Indicated proteins were transiently transfected into HEK293T cells for 48 h, and then cells’ lysis were applied for Western blot. Western blot scanning densitometry for three independent experiments. ns = none significance; ** *p* < 0.01; (**C**) HEK293T cells were first transfected with siRNA of FBXO22 for 24 h, and then cells were replated into 6-well plates overnight. Then, cells were treated with cycloheximide (100 µg/mL) for indicated time and cell lysis was applied for Western blot. A CD147 degradation curve was drawn according to the scanning densitometry ratio of CD147/α-tubulin. The initial ratio of CD147/α-tubulin is about 7 (red on y-coordinate) in both groups, and the cycloheximide treated time is indicated on x-coordinate following the dotted line when the ratio reached 3.5 (red on y-coordinate). ** *p* < 0.01; (**D**) knockdown of FBXO22 using 3 independent siRNAs increased CD147 levels in SMMC-7721, Huh-7 and A549 cells; (**E**) ectopically expressed FBXO22 led to a decrease of CD147 levels in SMMC-7721, Huh-7 and A549 cells; and (**F**) the mRNA level of CD147 was not influenced by FBXO22 in SMMC-7721, Huh-7 and A549 cells. * *p* < 0.05, ** *p* < 0.01, *** *p* < 0.001.

To investigate whether FBXO22 mediates the poly-ubiquitination and degradation of CD147, we first performed the poly-ubiquitination experiment. Figure 3A demonstrated that knocking down of FBXO22 inhibited the poly-ubiquitination of GFP-CD147 and CD147, while overexpression of FBXO22 promoted GFP-CD147 and CD147 poly-ubiquitination. Moreover, FBXO22 overexpression inhibits the expression of GFP-CD147, but not GFP-CD147-ΔICD in HEK293T cells (Figure 3B). We also found after knockdown of FBXO22 that the degradation rate of CD147 decreased, or the half-life of CD147 was prolonged (Figure 3C). Furthermore, after FBXO22 knockdown with siRNA in SMMC-7721, Huh-7 and A549 cells, the level of CD147 increased (Figure 3D). On the other hand, overexpressing FBXO22 downregulated CD147 level in the three cell lines above (Figure 3E). Meanwhile, the mRNA level of CD147 was not influenced by FBXO22 (Figure 3F), indicating that altered CD147 protein abundance by FBXO22 is primarily due to changes in protein turnover. To conclude, FBXO22 may mediate the poly-ubiquitination and degradation of CD147 by interacting with CD147-ICD.

### 2.4. FBXO22-CD147 Axis Is Involved in A549/DDP Chemo-Resistance

**Figure 4:**
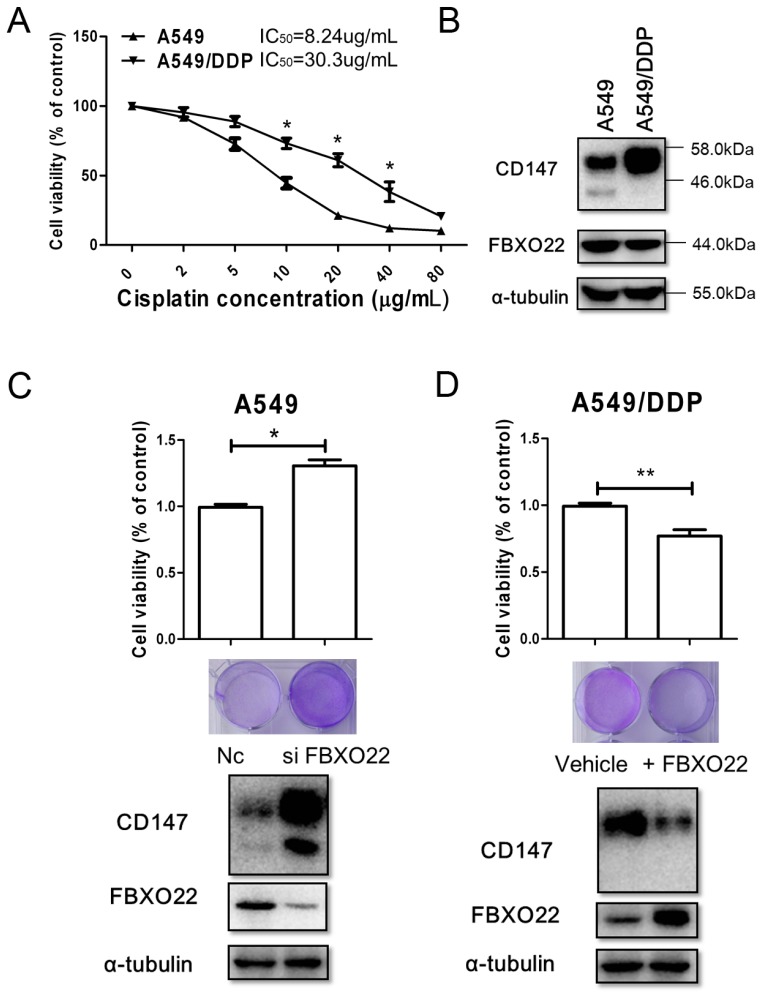
FBXO22-CD147 axis is involved in A549/DDP chemo-resistance. (**A**) Growing A549 and A549/DDP cells seeded in in 96-well plates at a density of 5 × 10^4^ cells/well and incubated overnight at 37 °C. Then cells were treated with increasing concentrations of cisplatin (0–80 µg/mL) for 48 h, and cell viability was assessed by MTT assay. * *p* < 0.05. The IC_50_ values of A549 and A549/DDP cells for cisplatin were also shown; (**B**) Representative Expression of CD147 and FBXO22 were detected in A549 and A549/DDP cells by Western blot; (**C**) FBXO22 knocking down in A549 cells enhanced cell resistance to cisplatin. * *p* < 0.05; (**D**) FBXO22 overexpression in A549/DDP cells sensitize cells to cisplatin. ** *p* < 0.01. Representative crystal violet staining results of cisplatin treated cells were also shown under the histograms in C and D.

CD147 is involved in the progression of chemotherapy resistance of cancer cells [6,7]. Figure 4A showed the sensitivity of A549 (wild-type NSCLC cells) cells and A549/DDP (cisplatin-resistant NSCLC cells) cells to cisplatin treatment. As expected, the level of CD147 was elevated in A549/DDP cells compared to A549 cells. Interestingly, increased CD147 was mainly highly glycosylated form (range from 46 to 58 kDa) while the lowly glycosylated form was undetectable (Figure 4B). Moreover, the protein level of FBXO22 in A549/DDP cells was decreased compared to A549 (Figure 4B). Next, the role of FBXO22 in the progression of chemotherapy resistance of A549/DDP cells was investigated. Clearly, knocking down FBXO22 promoted the survival of A549 cells treated with 40 μM cisplatin (Figure 4C), while FBXO22 overexpression conferred sensitivity of A549/DDP cells to cisplatin (Figure 4D).

## 3. Discussion

In the present study, we have demonstrated that internalized CD147 is a biological target of FBXO22, which was previously reported to interact with and target many substrates for proteasome-dependent destruction. The accumulation of highly glycosylated form CD147 in A549/DDP cells may attribute to its low expression of FBXO22. FBXO22 emerges as a critical gene conferring chemotherapeutic sensitivity to tumor cells.

After internalization through ARF6-related CIE, microtubule-dependent endosomal sorting by hook1, CD147 could be recycled back to the cell surface [10,18]. CD147-ICD plays a critical roles in the recycling of CD147 by interacting with hook1 directly [10]. Here, we found that both GFP-CD147 and GFP-CD147-ΔICD could be accumulated in the cytoplasm after co-transfection with constitutively activated ARF6, indicating that CD147-ICD is independent for CD147 internalization. This is an important characteristic to distinguish CD147, a CIE protein from CME (clathrin mediated endocytosis) proteins [21,22]. As CD147-ICD is indispensable for its recycling, it was observed that deletion of CD147-ICD in GFP-CD147 further enhances GFP-CD147-ΔICD accumulation in the cytoplasm. Generally, accumulated GFP-CD147-ΔICD was proposed to traffic into the default degradation pathway. However, the accumulated GFP-CD147-ΔICD seems more stable in HEK293T cells (Figure 1C,D).

Considering the existence of a balance between ubiquitination and deubiquitination dominating the destiny of internalized proteins, we hypothesized that GFP-CD147, rather than GFP-CD147-ΔICD, be ubiquitylated in HEK293T cells, resulting in the degradation of GFP-CD147. Then, FBXO22 was identified to interact with CD147-ICD, and FBXO22 mediates poly-ubiquitination and degradation of CD147 in SMMC-7721, Huh-7 and A549 cells. FBXO22 has also been reported to mediate the degradation of KDM4A, KLF4 and methylated p53 [15,16,17]. Previously, it was reported misfolded CD147 in endoplasmic reticulum is degraded through endoplasmic reticulum associated degradation (ERAD) pathway by a proteasome system [23,24]. However, the ubiquitination of misfolded CD147 (redundant product) would not directly influence the function of CD147. Indeed, TRAF6 regulates melanoma invasion and metastasis through ubiquitination of membrane-localized CD147 [9]. In addition, ubiquitination of membrane-localized CD147 by TRAF6 activates signaling pathways enhancing melanoma invasion and metastasis. Here, our study highlighted that FBXO22 could specifically recognize the CD147 at the intracellular domain, and enhance its polyubiquitination and degradation. This further diversifies the ubiquitination forms of CD147 and adds complexity to the CD147 regulation at the post-translational level. Clearly, the ubiquitination of CD147 described here competitively inhibits the recycling of CD147.

Traditional chemotherapy always suffers from the barrier of drug resistance owing to a small subset of cancer cells involved in chemotherapy resistance and tumor recurrence [1,2]. Previously, CD147 determines chemotherapy resistance by cooperating with HA-receptor or MCT1 (monocarboxylate transporter 1) [7,25]. In particular, CD147 was implicated in the chemo-resistance of cancer stem cells as elevated expression of CD147 was detected in CSC-like MDA-MB453 cells. Moreover, ARF6 is actually downregulated in CSC-like MDA-MB453 cells [6]. It is reasonable that downregulation of ARF6 inhibits the internalization of CD147 and thereafter CD147 is accumulated on the cell membrane. Here in this study, CD147 of highly glycosylated form was especially elevated in A549/DDP cells compared to wild type A549 cells (Figure 4B). Meanwhile, the expression of FBXO22 was downregulated in A549/DDP cells. As FBXO22 targets the internalized CD147 for degradation, more internalized CD147 would be recycled back to the cell surface without enough FBXO22 in A549/DDP cells. Therefore, accumulation of highly glycosylated form CD147 was observed in our results. Even though it is plausible that glycosylation of CD147 is enhanced in A549/DDP cells, our study here underlined that FBXO22-CD147 axis is involved in A549/DDP chemo-resistant. Furthermore, FBXL5, another F-box protein, was reported to attenuate RhoGDI2-induced cisplatin resistance in gastric cancer cells [26]. Future work that delineates the role of FBXO22 is expected to enhance our knowledge about the mechanisms of drug resistance.

## 4. Materials and Methods

### 4.1. Cell Culture and Reagents

Human hepatocellular carcinoma SMMC-7721 cells were purchased from Institute of Biochemistry and Cell Biology (Shanghai, China). Human hepatocellular carcinoma Huh-7 cells were obtained from Cell Bank of the JCRB (Tokyo, Japan). Human non-small-cell lung carcinoma (NSCLC) A549 and A549/DDP were from Cancer Institute of the Chinese Academy of Medical Sciences (Beijing, China). HEK293T cells were obtained from the American Type Culture Collection (ATCC, Manassas, VA, USA). SMMC-7721, A549 and HEK293T cells were cultured in RPMI1640 (Gibco, New York, NY, USA) with 10% fetal bovine serum, 2 mM glutamine, 100 U/mL penicillin, and 100 µg/mL streptomycin in 5% CO_2_ atmosphere at 37 °C. For the culture of A549/DDP, cisplatin (1 µg/mL) was added into RPMI1640 medium described above. Huh-7 cells were cultured in Dulbecco’s Modified Eagle Medium (DMEM) supplemented with 10% FBS. Cycloheximide (66-81-9) were from Cyman (Ann Arbor, MI, USA). Antibodies to GFP (sc-9996) and Ub (sc-271289) were from Santa Cruz Biotechnology (Dallas, TX, USA). Antibodies to FBXO22 (13606-1-AP) and GST (CW0286a) were from Proteintech (Wuhan, China) and CWBio (Beijing, China), respectively. Antibodies to CD147 (HAb 18, Ig G1) and α-tubulin were developed in our laboratory [27].

### 4.2. Plasmids

pGEX-6P-1 and pcDNA3.1 were from Invitrogen (Carlsbad, CA, USA), peGFP-N1 and peGFP-C2 were from Clontech (Mountain View, CA, USA). After sequence encoding, eGFP was inserted into the signal peptide and coding sequence of CD147 (NM_198589.2) by overlapping PCR (Polymerase Chain Reaction), and the generated sequence was fused into peGFP-N1 with Hind III/Not I to create GFP-CD147. GFP-CD147-ΔICD was generated after deleting the intracellular domain of GFP-CD147.The sequence coding for intercellular domain of CD147 (residues 230–269 of CD147) was cloned into peGFP-C2 or pGEX-6P-1 to produce eGFP-C2-ICD and pGST-CD147-ICD, respectively. FBXO22 (NM_147188.2) was cloned and inserted into pcDNA3.1 with standard PCR and cloning techniques.

### 4.3. Immunofluorescence

Immunofluorescence was performed as previously described [11]. Here in brief, growing SMMC-7721 cells (5 × 10^4^) were first seeded into to dishes pre-coated with 1% Matrigel (BD Bioscience, Franklin Lakes, NJ, USA) overnight. Then, cells were transfected with indicated plasmids for 36 h. The nuclei were counterstained with DAPI (Vector Labs, Burlingame, CA, USA). The samples were visualized with a confocal microscope using Nikon NIS-Elements software (Nikon, Tokyo, Japan).

### 4.4. RNA Interference and Transfection

Three FBXO22 siRNAs (GenePharma, Shanghai, China) targeting different regions of the FBXO22 transcript were used in this study [15]; FBXO22 siRNA_1, 5′-GCACCUUCGUGUUGAGUAA-3′; FBXO22 siRNA_2, 5′-GGUGGGAGCCAGUAAUUAU-3′; FBXO22 siRNA_3, 5′-GUUCGCAUCUUACCACAUA-3′. Control nonspecific siRNA of sense strand and antisense strand was 5′-UUCUCCGAACGUGUCACGUTT-3′ and 5′-ACGUGACACGUUCGGAGAATT-3′, respectively. The cells were transfected with the siRNAs or plasmids using Lipofectamine 2000 (Invitrogen, Carlsbad, CA, USA).

### 4.5. GST Fusion Protein Pull-Down Assay

pGST-CD147-ICD described above was transformed into BL21(DE3) *E. coli* cells (Novagen). Cells were grown in LB media at 37 °C and when cells with A600 = 0.6, recombinant fusion proteins were induced by adding isopropyl-1-thio-A-d-galactopyranoside (0.1 mM) for 12 h at 18 °C. Then, cells were harvested, washed with cold PBS and stored at −80 °C overnight. Cell pellets were re-suspended by cold PBS and disrupted by sonication before purification. The lysate was centrifuged (12,000× *g*, 30 min) and glutathione Sepharose 4 B gel (Amersham Biosciences, Piscataway, NJ, USA) was added to the supernatant followed by washing with PBS. The purified GST fusion protein was eluted with PBS containing saturated reduced glutathione. Similarly, purified GST tag was produced by transforming BL21 (DE3) with pGEX-6P-1 following the procedures mentioned above. A ProFound Mammalian co-IP Kit (Pierce Biotechnology, Rockford, IL, USA) was used to investigate the potential molecules interacting with the intracellular domain of CD147, according to the manufacturer’s instructions. Briefly, cells (1 × 10^7^) were lysed using IP Lysis/Wash Buffer. The lysate was collected onto a coupling resin pre-bound with 20 μg GST-CD147-ICD or GST, followed by four washes with IP Lysis/Wash Buffer. The coupling resin was then washed with elution buffer. The eluted samples were then applied to further analysis by mass spectrometry or Western blot. Western blot was performed as described previously [11]. Anti-mouse and anti-rabbit secondary antibodies conjugated to horseradish peroxidase were from Pierce.

### 4.6. Mass Spectrometry and Western Blot

IP-mass spectrometry was performed as previously described [28]. The IP elution was restored with 10 mM dithiothreitol (DTT), digested overnight with trypsin (90055, Mass Spectrometry-Grade Endoproteinases, Thermo Scientific, Pittsburgh, PA, USA) in a ratio of 1:20. Digestion was stopped with 10 mM DTT and the peptide solution was desalted with C-18 Tips (87784, Thermo Scientific) following the manufacturer’s instructions. Then, samples were analyzed on an LTQ-Orbitrap XL ETD mass spectrometer (Thermo Finnigan, San Jose, CA, USA). Peptides were identified using Sequest (ThermoQuest, San Jose, CA, USA).

### 4.7. Ubiquitination Assay

Forty hours posttransfection, HEK 293T cells expressing GFP-CD147 were washed with PBS supplemented with 200 μM iodoacetamide and 10 mM *N*-ethylmaleimide (NEM; Sigma, St Louis, MO, USA). Cells were lysed with RIPA lysis buffer (150 mM NaCl, 20 mM Tris-HCl pH7.4, 5 mM EDTA, 1% NP-40, 1% Na-deoxychol ate, 0.1% SDS (sodium dodecyl sulfate), 1 mM PMSF (phenylmethanesulfonyl fluoride), 20 μg/mL Leupeptin, 20 μg/mL Aprotini, 3 μg/mL Pepstatin A) on ice for 30 min. Cleared lysates were quantified, and an equal amount of each lysate was used for immunoprecipitation with coupling resin (26149, Pierce, Rockford, IL, USA) pre-bound with antibody to Ub (sc-271289, Santa, Dallas, TX, USA). Resins were washed with lysis buffer, and samples were eluted. Elution buffer was further administrated on an SDS-PAGE (polyacrylamide gel electrophoresis) gel. Subsequent immunoblot was performed using GFP antibody and CD147 antibody mentioned above.

### 4.8. qRT-PCR

Total RNA extraction and qRT-PCR were performed as previously described [11]. The following primers were used in this study: GAPDH: Forward 5′-GCACCGTCAAGGCTGAGAAC-3′, Reverse 5′-TGGTGAAGACGCCAGTGGA-3′; FBXO22: Forward 5′-CTATGCTGGCGTAATCGGGT-3′, Reverse 5′-ATACTCTGCGCACACACTCC-3′; CD147: Forward 5′-ACTCCTCACCTGCTCCTTGA-3′, Reverse 5′-GCCTCCATGTTCAGGTTCTC-3′.

### 4.9. Cell Viability Assay

The sensitivity of A549 and A549/DDP cells to cisplatin was determined as previously described [29]. Briefly, after cells (5 × 10^4^ cells/well) were seeded in 96-well plates and incubated overnight at 37 °C, cells were incubated with different concentrations of cisplatin (0, 2, 5, 10, 20, 40 or 80 µg/mL) for 48 h. Then, a standard MTT assay was performed to determine the cell viability. As to the assay of FBXO22 sensitize cells to cisplatin treatment, FBXO22 was first knocked down in A549 cells, or FBXO22 overexpressed in A549/DDP cells. Transfected cells were then seeded into 96-well plates as described above and cells were incubated with 40 µg/mL cisplatin for 48 h. Finally, the cell viability of transfected cells were analyzed by MTT and crystal violet (0.2%) staining.

### 4.10. Statistical Analysis

Data are presented as mean ± SD. Statistical analysis between groups was performed with GraphPad Prism v5.0 software (GraphPad Software, La Jolla, CA, USA) using an unpaired Student’s *t*-test and one-way ANOVA. Differences with *p* < 0.05 were considered statistically significant.

## 5. Conclusions

To conclude, our study here demonstrated that FBXO22 mediates polyubiquitination and degradation of CD147 by interacting with its intracellular domain. Downregulation of FBXO22 disturbed the balance of recycle and degradation of CD147, whose accumulation contributed to the cisplatin resistance of A549/DDP cells. This work further confirmed CD147 as an important target for tumor treatment.
